# Intersect-then-combine approach: improving the performance of somatic variant calling in whole exome sequencing data using multiple aligners and callers

**DOI:** 10.1186/s13073-017-0425-1

**Published:** 2017-04-18

**Authors:** Maurizio Callari, Stephen-John Sammut, Leticia De Mattos-Arruda, Alejandra Bruna, Oscar M. Rueda, Suet-Feung Chin, Carlos Caldas

**Affiliations:** CRUK Cambridge Institute, University of Cambridge, Cambridge, UK

**Keywords:** Somatic mutation, Variant calling, Whole exome sequencing, NA12878, Platinum genome, Mutect2, Strelka, BWA, Novoalign, Filtering

## Abstract

Bioinformatic analysis of genomic sequencing data to identify somatic mutations in cancer samples is far from achieving the required robustness and standardisation. In this study we generated a whole exome sequencing benchmark dataset using the platinum genome sample NA12878 and developed an intersect-then-combine (ITC) approach to increase the accuracy in calling single nucleotide variants (SNVs) and indels in tumour-normal pairs. We evaluated the effect of alignment, base quality recalibration, mutation caller and filtering on sensitivity and false positive rate. The ITC approach increased the sensitivity up to 17.1%, without increasing the false positive rate per megabase (FPR/Mb) and its validity was confirmed in a set of clinical samples.

**Electronic supplementary material:**

The online version of this article (doi:10.1186/s13073-017-0425-1) contains supplementary material, which is available to authorized users.

## Background

The rapid development of high-throughput sequencing (or next generation sequencing (NGS)) technology has enabled great progress in cancer genomics. Decreases in costs and increases in data output have resulted in the systematic collection of genome-scale data in large tumour cohorts [1, 2], improving our understanding of the mechanisms underlying tumour development, progression and response to treatments, in addition to setting the foundation for precision medicine [3, 4]. However, in contrast with the increasing ease in data generation, bioinformatic analyses have yet to reach a satisfactory level of robustness and standardisation, both of which are essential for correct data interpretation and eventual clinical translation [5].

Currently, whole exome sequencing (WES) offers the best trade-off between costs and amount of genetic information obtained for detecting single nucleotide variants (SNVs) and small insertions and deletions (indels) in coding regions. Identifying somatic mutations is more challenging than identifying germline variants for several reasons: (1) tumour samples can contain a high amount of normal tissue contamination, (2) tumour cells can have acquired major changes in ploidy and DNA copy number and, finally, (3) somatic mutations can be present in a subset of tumour cells (i.e. subclonal mutations). As a consequence, lower variant allele frequencies (VAFs) need to be detected, making them harder to distinguish from technical noise [5].

A number of methods have been designed specifically to identify somatic mutations. Several studies comparing their performances [6–8] have highlighted poor concordance between methods [5, 9, 10]. In addition, not only the mutation caller, but also all the upstream computational steps can impact the final results. Unfortunately, identifying the best approach or appropriate parameters is extremely challenging because the ground truth is normally unknown.

An important benchmark dataset has been generated by the Platinum Genomes project, where a catalogue of highly accurate whole genome variant calls and homozygous reference calls has been derived for sample NA12878 by integrating independent sequencing data and the results of multiple pipelines (https://www.illumina.com/platinumgenomes). For example, it is the basis of the Genome Comparison and Analytic Testing (GCAT) platform [11] that allows an easy benchmarking of user’s pipelines for the identification of germline variants.

To generate a benchmark dataset for the detection of ‘somatic’ mutations, we performed a WES experiment using two lymphoblastoid cell lines from the HapMap/1000 Genomes Project (NA12878 and NA11840) in order to mimic a tumour-normal pair, with NA12878 being the ‘tumour’ and NA11840 being the ‘normal’. We diluted the NA12878 DNA with an increasing amount of NA11840 DNA (from 0 to 99.8%) to estimate the performance in detecting mutations within a wide range of VAFs. Using this dataset we aimed to: (1) evaluate the effect of alignment and base quality recalibration on mutation calls; (2) compare the performance of Mutect2 and Strelka in identifying SNVs and indels; (3) optimize the mutation calling by parameter adjustment; (4) derive an ‘intersect-then-combine’ (ITC) approach to merge information from multiple tools to increase the sensitivity and decrease the false positive rate (FPR). The validity of the ITC approach was then confirmed in a set of clinical samples.

## Methods

### Sample preparation

Two lymphoblastoid cell lines, NA12878 and NA11840, from the Human Genome Diversity Project (HGDP)-CEPH collection were obtained from the Coriell Cell Repository. The NA11840 cell line was chosen from a set of 17 available CEPH cell lines in our laboratory as it shared the least number of SNVs with NA12878, so as to generate the maximum number of virtual somatic SNVs. The cell lines were grown as suspensions in RPMI 1640-Glutamax (Invitrogen, Waltham, MA, USA) supplemented with 10% foetal calf serum and 5% penicillin and streptomycin at 37 °C and 5% CO_2_. The cell lines were passaged at 1:10 dilution, and 10 × 10^6^ cells were harvested for DNA extractions.

DNA was extracted from the cell lines using the DNeasy Blood and Tissue DNA extraction kit (Qiagen, Manchester, UK) and quantified using a Qubit High Sensitivity DNA quantification kit (Life Technologies, Carlsbad, CA, USA). DNA from both cell lines was diluted to obtain 100 ng/μl stock concentrations.

To generate the serial dilutions of one cell line with the other, we mixed by volume to obtain the percentage (volume/volume) as presented in Additional file 1 (*n* = 12).

### Clinical samples

We included WES data from peripheral blood lymphocytes (‘buffy coat’) of 10 individuals. The samples were collected and analysed as part of the study ’Cell-free DNA in non-metastatic setting’, approved by the Institutional Review Board of the Vall d’Hebron University Hospital, Barcelona, Spain (PR_AG_67-2013). DNA was extracted using the QIAamp DNA Mini Kit (Qiagen) according to manufacturer’s instructions and quantified using the Qubit Fluorometer assay (Life Technologies) as previously described [12]. Two (or three) independent libraries were generated from each sample and sequenced as described in the next paragraphs. These libraries were sequenced on an Illumina HiSeq 2500 and Illumina HiSeq 2000 respectively (Illumina, San Diego, CA, USA).

A breast cancer sample for which two independent WES data were available was also included. This sample (ID: AB551) is part of a previously reported biobank [13].

### Whole exome sequencing

Adapter-ligated indexed libraries were generated using the Illumina Nextera Rapid Capture kit (Illumina) from 50 ng of DNA as per manufacturer’s instructions. The libraries were quantified using a Qubit High Sensitivity dsDNA assay (Life Technologies). Five hundred nanograms of adapter-ligated barcoded DNA from each sample from each library were pooled into a capture pool of 12. Each capture pool was hybridised twice with enrichment probes for the exome. The fragment sizes of enriched libraries were assessed using a bioanalyser (Agilent Technologies, Folsom, CA, USA) and quantified using KAPA Library Quantification Kits (Kapa Biosystems, Wilmington, MA, USA).

Paired-end 125-bp sequencing runs were performed on an Illumina HiSeq 2500 instrument, aiming for a mean read depth coverage of 100 for the NA12878 dilution series.

### Alignment

#### Burrows-Wheeler Aligner (BWA)/Genome Analysis Toolkit (GATK) pipeline

After initial quality control using the FastQC application (http://www.bioinformatics.babraham.ac.uk/projects/fastqc/), adapters and low-quality bases (Phred score below 20) were trimmed off using Trim Galore (v0.3.7) (http://www.bioinformatics.babraham.ac.uk/projects/trim_galore/). Reads were aligned to the human reference genome (hg19/GRCh37 decoy) using BWA-MEM (v0.7.12) and default parameters. Local realignment and base quality recalibration (BQR) were performed using the GATK, v3.4.46. Multiple bam files for the same sample (obtained on different sequencing lanes) were merged. Alignment and coverage metrics as well as PCR duplicate marking were computed using Picard tools (v1.125) before and after merging. Local realignment was repeated on all 12 samples together to ensure indel concordance between samples.

#### Novocraft pipeline

Adapter and low-quality base (Phred score below 20) trimming, alignment and BQR were performed in a single step using Novoalign (v3.02). In a preliminary step, the alignment score threshold was varied between 50 and 300. As shown in Additional file 2: Figure S1, modification of this threshold affects both alignment efficiency and the running time, and we established that the best compromise between alignment time and efficiency was at a score threshold of 250. Bam files were locally realigned using the GATK, v3.4.46. Sorting and duplicate marking were done using Novosort (v3.02) before and after merging the distinct bam files from the same sample. Local realignment was repeated after merging on all 12 samples together.

### Identification of regions of interest

Platinum variant calls for sample NA12878 (the virtual ‘tumour’) and confident regions (high confidence homozygous reference regions plus platinum calls) [14] were downloaded from https://www.illumina.com/platinumgenomes (v7.0.0). Genotype data for sample NA11840 (the virtual ‘normal’) were obtained from the 1000 Genomes website. Platinum calls were intersected with the Nextera exome target regions, and variants shared with the NA11840 sample were excluded. Four multiallelic SNVs and 10 multiallelic indels were also excluded. In total we identified a list of 9968 SNVs and 420 indels that are theoretically ‘somatic variants’ in our virtual tumour-normal pair. The confident regions were also intersected with the Nextera exome target regions, defining the regions of interest in which to search for mutations. After subtracting platinum call regions, a total of 36,582,697 bp represented our set of reference regions.

### Somatic mutation calling

#### Mutect2

Mutect2 (included in GATK 3.5) was run for each combination of NA12878 dilution series (from 100% to 0.2% purity) with the NA11840 sample (tumour-normal mode) using default parameters, with the exception of the *minPruning* parameter which determines the minimum support to not prune paths in the De Bruijn-like graph: this was set at 3 (instead of the default value 2), as it dramatically reduced the running time without affecting the number of variant calls (data not shown). In the platinum genome dilution experiment, we included all mutations passing all the internal filters as well as mutations that failed the ’clustered_events’ and/or ’homologous_mapping_event’ filters, as germline variations frequently occur close to each other in the genome.

#### Strelka

We ran Strelka (v1.0.14) [15] for each virtual ‘tumour-normal’ pair with recommended starting parameters for BWA in the configuration file and default parameters. The *isSkipDepthFilters* parameter was set to 1 to skip depth filtration, as suggested by the authors. Mutations called by default were those passing internal filters identified using Tier 1 reads and with a QSS_NT > 15 for SNVs and a QSI_NT > 30 for indels.

### Performance evaluation

By intersecting the experimental calls with the list of platinum calls (9968 SNVs and 420 indels), we computed the number of true positives (TP), false negatives (FN) and false positives (FP). This task was performed using custom scripts that matched the genomic coordinates as well as reference and alternative alleles. Sensitivity was then defined as:

$$ Sens = \frac{TP}{TP + FN} $$

We also computed the false positive rate per megabase (FPR/Mb) as:

$$ F P R/ Mb=\frac{FP}{n\; ref}\times {10}^6 $$

where *n ref* is our set of reference regions equal to 36,582,697 bp.

### Variant annotation

Somatic mutations called in the breast cancer clinical sample were annotated using Variant Effect Predictor (VEP) (http://grch37.ensembl.org/).

## Results

### Experimental design and quality control

We sequenced two cell lines from the HapMap/1000 Genomes Project, NA12878 (Platinum Genomes) and NA11840 to mimic a tumour-normal pair allowing identification of ‘somatic’ SNVs and indels. The NA12878 sample was also mixed with an increasing amount of NA11840 (up to 99.8% by concentration, Additional file 1) to mimic normal contamination and the presence of subclonal somatic mutations. All samples were subjected to WES, and we obtained an average on target coverage of 100× (Additional file 2: Figure S2A).

A list of 9968 high confidence SNVs present in NA12878 and not in NA11840 was derived (see Methods). For these loci we computed the VAFs in our data to verify that the observed median values for heterozygous and homozygous SNVs matched the expected values in the dilution series (Additional file 2: Figure S2B). It is worth noting that whilst the median VAFs matched the expected values, confirming dilution accuracy, a wide dispersion of VAF values was observed, with 265 SNVs having a VAF of 0 in the 100% NA12878 sample, mostly caused by low or no coverage in that specific locus. An additional group of 495 SNVs had coverage less than 10× in our dataset. We did not exclude these SNVs, as uneven coverage is commonly observed in WES data. Although the selected platinum calls were not identified as SNVs in the publicly available data for NA11840, some were clearly present in our NA11840 data (556 mutations had a VAF greater than 0.2, Additional file 2: Figure S3). Again, these loci were retained because they might be either real SNVs or problematic regions with higher noise. The inclusion of the above-mentioned SNVs meant that our sensitivity would never reach 100%.

**Fig. 1:**
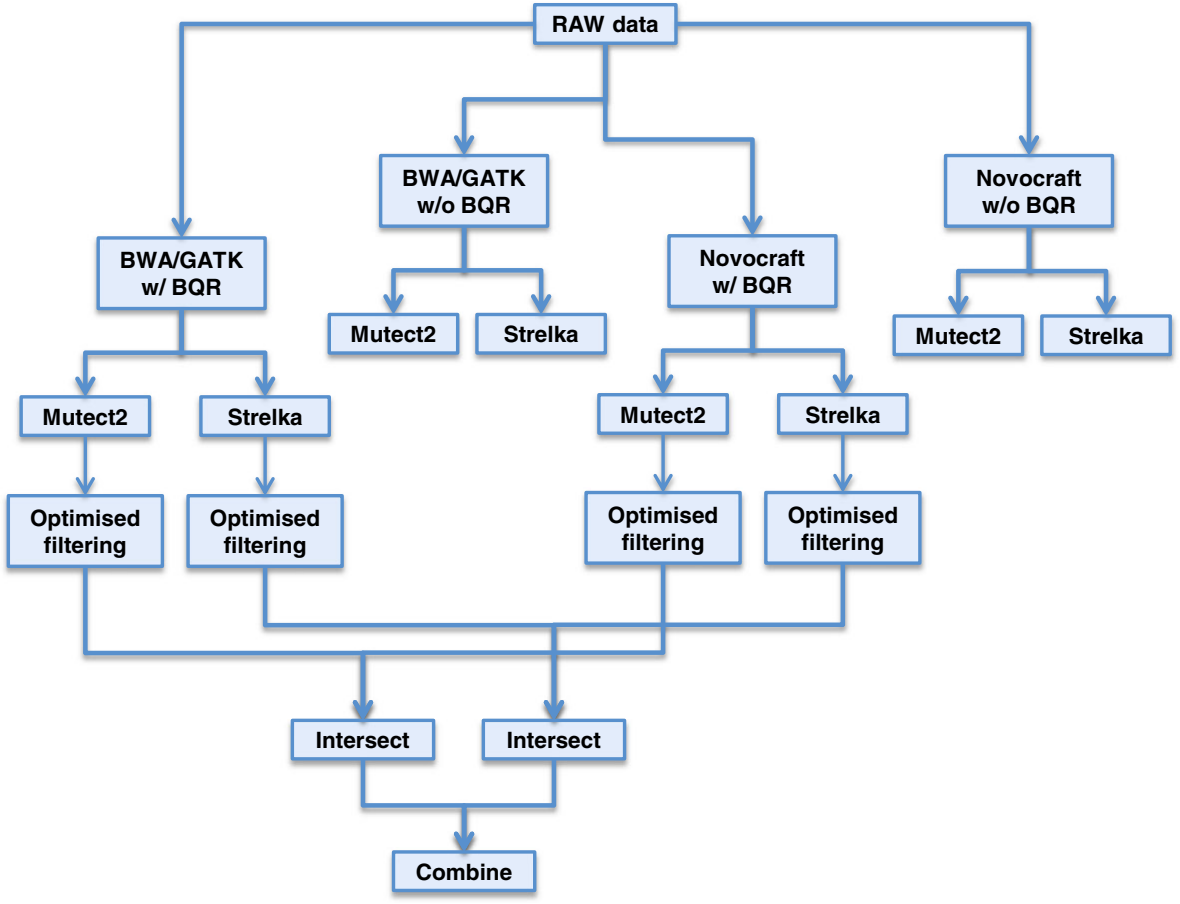
Analysis workflow. Schematic representation of the workflow of analyses applied to the NA12878 platinum genome dilution series. Raw data have been initially analysed using two alignment pipelines (BWA/GATK-based and Novocraft-based) with or without base quality recalibration. Somatic calls were identified using two distinct tools: Mutect2 and Strelka. For base quality recalibrated data, mutation caller parameters have been adjusted to improve overall performance. The intersection between mutations from the same caller but different alignment pipelines was selected to reduce the number of false positives. Subsequently, the unions of these filtered calls from Mutect2 and Strelka were combined to obtain the final set of calls

Raw sequencing data were processed using two distinct alignment pipelines (hereafter named BWA/GATK and Novocraft; the statistics above have been computed on Novocraft bam files), and ‘somatic’ SNVs and indels were called using Mutect2 and Strelka. Mutations were initially identified using default parameters and again using optimised filtering criteria. We then applied an intersect-then-combine (ITC) approach as detailed in Fig. 1 and subsequent paragraphs.

Performances of the different pipelines were measured in terms of sensitivity computed for each sample in the dilution series and FPR/Mb estimated in the most diluted sample. For FPR/Mb estimation, we only used the most diluted sample because we observed new mutations in the least diluted NA12878 samples (likely to be caused by genetic drift) that would be wrongly accounted as false positives. This is supported by the fact that in the least diluted samples (100% and 80% NA12878) most of the hypothetical false positives overlap, whilst in the most diluted samples none of them overlaps (Additional file 2: Figure S4).

### Mutation calling using default parameters and effect of base quality recalibration

**Fig. 2:**
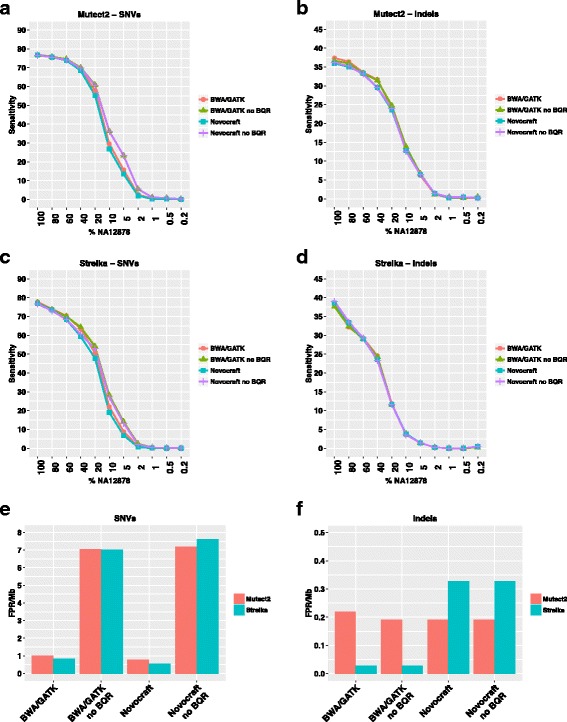
Effect of alignment and base quality recalibration on sensitivity and FPR. Sensitivity in detecting SNVs (**a**, **c**) or indels (**b**, **d**) in our dilution series using Mutect2 (**a**, **b**) or Strelka (**c**, **d**) and each of four different alignment pipelines. **e**, **f** FPR/Mb in SNV (**e**) or indel (**f**) calling as a function of the alignment pipeline and mutation caller used

When calling SNVs using default parameters, Mutect2 had higher sensitivity, particularly at low VAFs, but at the expense of a slightly higher FPR/Mb when compared to Strelka (Fig. 2a, c, e). However, none of the approaches reached 80% sensitivity in the pure NA12878 sample. Performances were lower in indel calling, with Strelka showing the lowest sensitivity but also a much lower FPR/Mb in combination with the BWA/GATK pipeline (Fig. 2b, d, f).

The base quality recalibration (BQR) step can considerably change the performance. Indeed, when BQR was not performed, low frequency SNVs were called with a higher sensitivity, but associated with a dramatic increase in FPR/Mb (Fig. 2a, c, e). Therefore, for all subsequent analyses, base quality recalibrated data were used. In base quality recalibrated data, although the alignment algorithms did not seem to have a major impact on sensitivity (Fig. 2a and c), the SNV FPR/Mb was higher in both callers when used with BWA/GATK alignments (Fig. 2e). In addition, a higher FPR/Mb was observed when Strelka was used to call indels on Novocraft-aligned data (Fig. 2f).

### Optimising mutation caller parameters

To improve the performance of both mutation callers, various caller-specific filtering criteria were assessed. In Mutect2, we looked at the reasons for rejection of the false negative calls to understand which internal filters caused rejection, thereby identifying which parameters needed adjustment (Additional file 2: Figure S5). We considered both the undiluted NA12878 and the 10% diluted sample, hypothesising that reasons for failure might be different at high and low VAFs. Indeed, in the undiluted NA12878, independent of the alignment pipeline, most of the false negatives were rejected because the alternative alleles were observed in the normal sample (i.e. NA11840). As previously mentioned, some of these false negatives might be due to the presence of unreported SNVs in the NA11840 sample; however, a proportion is likely caused by background noise or technical cross-sample contamination (Additional file 2: Figure S3B).

Default Mutect2 parameters for the ‘alt_allele_in_normal’ filter allow for no more than one read bearing the alternative allele in the normal sample, and it must represent less than 3% of the reads mapping over the locus. Taking advantage of a dataset where the ground truth (or a good approximation of it) is known, we measured the change in performance as a function of the threshold applied. Increasing the percentage of alternative allele present in the normal sample will likely increase not only the sensitivity but also the FPR, mainly because one might call germline mutations as somatic. Therefore, we added an extra filter by computing the ratio between the VAF observed in the tumour and the VAF observed in the normal (hereafter called T/N ratio). As expected, increasing the percentage of alternative allele allowed in the normal increased both sensitivity and FPR/Mb, but by applying the T/N ratio we could obtain higher sensitivity and lower FPR/Mb compared with default parameters. This was true for both 100% and 10% NA12878 samples and independent of the alignment pipelines (Additional file 2: Figures S6, 7).

Not surprisingly, in the 10% NA12878 sample the main reason of failure was the ‘t_lod_fstar’ filter, where the log-likelihood ratio of the data under the variant and reference models has to exceed a specified threshold (default = 6.3). Varying the threshold confirmed that it is a trade-off between sensitivity and FPR/Mb (Additional file 2: Figures S6, 7).

In Strelka, mutation calls are separated in Tier 1 and Tier 2, where the first is a set of input data filtration and model parameter settings with relatively stringent values, whereas the second uses more permissive settings [15]. It has been suggested by the Strelka authors to consider Tier 1 calls only and apply a QSS_NT (quality score reflecting the joint probability of somatic variant and genotype of the normal) threshold of 15. Including Tier 2 calls increased both sensitivity and FPR/Mb, but by also changing the QSS_NT threshold it was possible to increase the sensitivity and decrease the FPR/Mb. As before, this is true independently of the dilution and alignment pipeline (Additional file 2: Figure S8). The same applies to indels, where the default QSI_NT threshold is 30, although smaller improvements were observed (Additional file 2: Figure S9).

**Fig. 3:**
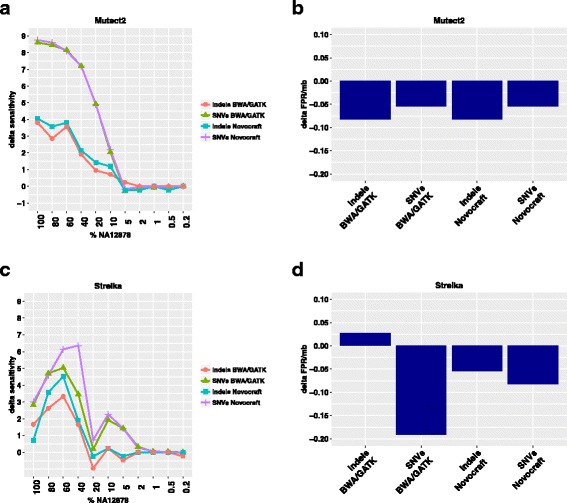
Effect of parameter adjustments on sensitivity and FPR. Changes in sensitivity (**a**, **c**) and FPR/Mb (**b**, **d**) after optimised filtering of Mutect2 (**a**, **b**) or Strelka (**c**, **d**) calls in combination with the two alignment pipelines (BWA/GATK and Novocraft)

Based on the analyses above, we selected the best thresholds to increase the sensitivity (in particular at high VAF, without losing sensitivity at low VAFs), whilst in most cases reducing the FPR/Mb. For Mutect2 we included SNVs and indels with a percentage of alternative allele in normal up to 7% but a T/N ratio higher than 5. In Strelka we included Tier 1 and Tier 2 calls with a QSS_NT > 25 for SNVs and QSI_NT > 35 for indels. The improvement in performance using the aforementioned parameters is summarised in Fig. 3.

### The intersect-then-combine (ITC) approach

After parameter optimisation, we hypothesised that some of the false positives might be caused by alignment errors, and to test this hypothesis we compared the calls from the same mutation caller but from different alignment pipelines. Interestingly, only a small percentage (an average of 1% for SNVs and 3.9% for indels) of true positive calls was discordant, whilst half of the false positives were called in one case but not the other (an average of 50% for SNVs and 48% for indels) (Table 1). Therefore, considering only the intersection of calls identified by the same mutation caller but with two different alignment pipelines reduced the sensitivity slightly but reduced the FPR dramatically. Importantly, this makes it possible to combine the calls from the two mutation callers (i.e. Mutect2 and Strelka), significantly increasing the sensitivity (because they still show a significant disagreement) but still at a low enough FPR. Notably, the subset of mutations called by both Mutect2 and Strelka has an extremely high true positive rate (Table 2). We checked that variants called by one caller but not the other were not caused by a different representation of the same variant. We found that only in one case the same insertion next to a repetitive region was represented as 14:29261307 A- > AC in Mutect2 and 14:29261305 A- > AAAC in Strelka.

**Table 1:** Intersection of calls obtained using different alignment pipelines

		TP - 100% NA12878^a^			FP		
		BWA/GATK only (%)	Intersection	Novocraft only (%)	BWA/GATK only (%)	Intersection	Novocraft only (%)
SNVs	Mutect2	90 (1.1)	8435	91 (1.1)	23 (65.7)	12	15 (55.6)
	Strelka	107 (1.3)	7928	37 (0.5)	12 (50.0)	12	5 (29.4)
Indels	Mutect2	11 (6.4)	162	6 (3.6)	3 (60.0)	2	2 (50.0)
	Strelka	7 (4.0)	169	3 (1.7)	0 (0.0)	2	8 (80.0)

^a^Out of 9968 candidate SNVs and 420 true positive

**Table 2:** Union of somatic mutations identified using different callers

	TP - 100% NA12878			FP		
	Mutect2 only	Intersection	Strelka only	Mutect2 only	Intersection	Strelka only
Union SNVs	712	7723	205	8	4	8
Union indels	42	120	49	2	0	2

**Fig. 4:**
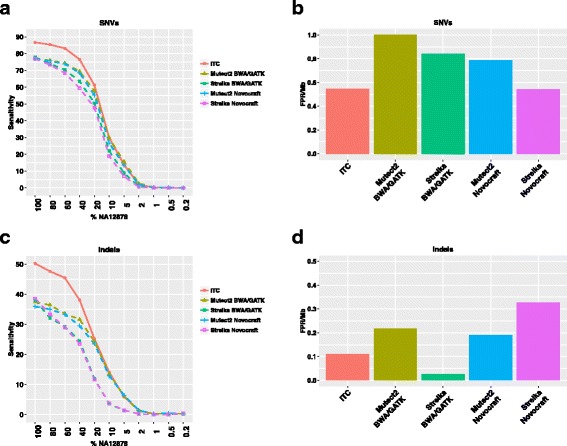
Performance of the intersect-then-combine (*ITC*) approach. Sensitivity (**a**) and FPR/Mb (**b**) in identifying somatic SNVs after applying the ITC strategy compared with performance using each single alignment pipeline in combination with each mutation caller. Sensitivity (**c**) and FPR/Mb (**d**) in identifying somatic indels after applying the ITC strategy compared with performance using each single alignment pipeline in combination with each mutation caller

The ITC approach allowed us to achieve a sensitivity of 86.7% for SNV calling in the pure sample and 50.2% sensitivity for indels; these values were significantly higher than the default performances for each single approach whilst simultaneously controlling for the FPR/Mb (Fig. 4). For example, the ITC approach showed the same FPR/Mb of the Strelka/Novocraft pipeline; however, the sensitivity was systematically higher across the dilution series. In particular, sensitivity in SNV detection increased up to 17.1% in the 40% NA12878, whilst sensitivity in indel detection increased up to 16.4% in the 60% NA12878 sample.

Sensitivity was also estimated for the subset of SNVs and indels heterozygous in the NA12878 sample (Additional file 2: Figure S10). This allows a VAF approximation for these SNVs and indels for each sample in the dilution series, hence allowing an estimation of the sensitivity as a function of the VAF. In this subset of mutations, the sensitivity in the 100% NA12878 sample (expected VAF = 50%) was 87.8% for SNVs and 55.4% for indels.

We anticipated a significant number of false negatives caused either by low coverage or alternative allele dropout (see the ’Experimental design and quality control’ subsection). Indeed, we found that 28.6% of the false negative SNVs using the ITC approach were due to low coverage (<10×) whilst an additional 9.9% showed a VAF = 0 in our data (but coverage >10×).

Finally, we looked at the false positive calls still present using the ITC approach (Additional file 2: Figure S11 and Additional file 3). Some of the false positive SNVs were clustered and could be avoided using extra filtering steps based on the distance between calls. In addition, we noticed the presence of several C > A transversions, probably caused by oxidative DNA damage during sample preparation [16]. False positive indels were located in low complexity/repetitive regions where polymerase slippage could introduce errors.

### Validation in clinical samples

**Fig. 5:**
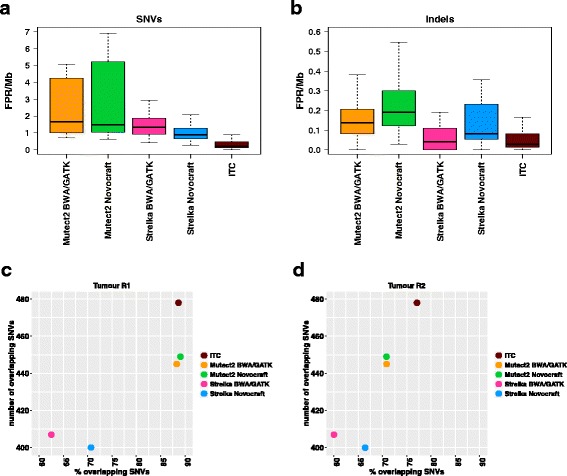
Validation in clinical samples. In 10 normal samples for which two or three sets of WES data from independent libraries were available we ran the different pipelines according to Fig. 1 using one replicate as ’tumour’ and the other as ’normal’ and vice versa, for a total of 28 comparisons (**a**, **b**). In this setting, all called mutations are treated as false positives. Boxplots represent FPR/Mb distributions for the 28 comparisons as a function of the different pipelines applied to identify somatic SNVs (**a**) or indels (**b**). The same set of analysis pipelines was applied to a breast cancer tumour sample for which two sets of WES data from independent libraries (R1 and R2) as well as matched normal WES data were available (**c**, **d**). The percentage (*x*-axis) and the total number (*y*-axis) of overlapping calls in the two replicates are plotted in **c** for R1 and **d** for R2. Sequencing coverage was 52× for R1 and 79× for R2

After developing our approach and testing the performance in the platinum genome experiment, we aimed to confirm its validity in clinical samples. We first used a set of 10 normal samples for which two or three independent replicates were available. We called ‘somatic’ mutations in each pair of replicates using one replicate as ‘tumour’ and the other as ‘normal’ and vice versa. In this setting, we assumed that any mutations called are false positives, giving us the opportunity to estimate the FPR/Mb. For the four combinations of alignment pipelines and mutation callers, the observed FPR/Mb values were slightly higher than what was observed in the benchmark dataset. Importantly, the FPR/Mb was the lowest using the ITC approach (Fig. 5a, b) and slightly lower than what was observed in the benchmark dataset (Fig. 4b, d).

Next, we looked at a tumour-normal pair for which two independent replicates of the tumour sample were available. In this case, mutations called in both cases are very likely to be true positives, while those not overlapping will be enriched in false positives. Consequently, an improvement in mutation calling performances should lead to an increase in the percentage and absolute number of overlapping mutations. Importantly, one replicate (R1) had a 52× average coverage whilst R2 had a 79× average coverage; this implies that most of the calls in R1 should overlap the calls in R2, whilst a higher percentage of non-overlapping calls can be expected for R2. For each replicate we computed the total number and the percentage of overlapping somatic mutations after applying each combination of alignment pipeline and mutation caller or the ITC approach. The observed pattern fits with the difference in coverage between the two replicates and, more importantly, shows that the ITC strategy leads to the highest number of overlapping mutations (Fig. 5c, d). Indeed, 37 extra mutations (overlapping between the two replicates) were identified with our approach compared with the second best (Mutect2 Novocraft). Interestingly, some of them were affecting cancer-related genes, i.e. a missense mutation in *TFE3*, a stop-gain mutation in *KMT2C* and a stop-gain mutation in the putative tumour suppressor gene *RPS6KA2* [17] (Additional file 4).

## Discussion

Cancer genomics has acquired a prominent role in oncology, providing information on cancer biology and mechanisms of resistance, and its clinical application is becoming a reality [18, 19]. However, computational analysis of sequencing-based data is facing a lack of standardisation, as demonstrated by recent reports [5, 20]. In this study we focused on improving the identification of somatic SNVs and indels in WES data.

Generating appropriate benchmark datasets to estimate pipeline performance is not a trivial task. In recent reports, somatic mutations have been spiked in computationally [20] or derived after manual curation of high coverage data [5]. The first approach is limited to SNVs and overestimates the performances because it generates mutations only in regions with sufficient coverage. The second is likely to generate an incomplete list of real mutations, in particular those having low frequency. The best available standard for germline mutation caller benchmarking is represented by the platinum genome sample NA12878 [11, 21]. Using this sample, we created here a benchmark dataset suitable for the evaluation of new methods and pipelines aiming to identify somatic mutations. A similar approach has been proposed in [6], but we generated a dilution series experimentally and not *in silico*, mimicking more realistically the detection of low VAF mutations. Although not a perfect system, we believe it represents one of the best possible approximations to the ground truth. Limitations are linked with the fact that tumour genomes are more complex than normal lymphoblastoid cell lines, and some low complexity or repetitive genomic regions might not have been considered. Remarkably, we were able to confirm our findings in clinical samples, obtaining similar FPR/Mb values and evidence for an increase in sensitivity using our ITC approach.

Note that, among mutations called with our approach and not with the second best performing pipeline, there were several missense mutations in proliferation and cancer genes, among them a stop-gain mutation in *RPS6KA2*, a putative tumour suppressor gene [17], and a stop-gain mutation in *KMT2C*, which has been found mutated in several cancer types (http://cancer.sanger.ac.uk/cosmic). This highlights how the bioinformatic analysis can significantly impact downstream data interpretation and the chances to identify the functionally relevant aberrations in a tumour sample.

In many studies, precision and recall are usually computed as metrics to estimate performance [5, 20]. We preferred to use sensitivity and FPR/Mb instead [6]. Precision, also known as true positive rate, is highly dependent on the number of real mutations in the sample. In our platinum genome experiment, nearly 10,000 mutations could be called, keeping the precision high even in the presence of hundreds of false positives. By contrast, the FPR/Mb gives a direct estimation of the expected number of false positives, independently of the number of real mutations present. Sensitivity and recall indicate the same metrics. Although the number of mutations in our benchmark dataset is higher than in most cancer types, this is not a shortcoming. On the contrary, from a statistical point of view, a higher number of candidate mutations helps in obtaining a more robust estimation of sensitivity.

We chose to use tools that have been shown to outperform others and are commonly used by the community and big cancer genomics consortia [5, 6]. Mutect2 has been recently released; therefore, the previous version has been more widely used and benchmarked. We present here the results obtained using Mutect2 because it has not been previously compared with other tools and also because of its ability to call both SNVs and indels. However, similar conclusions can be drawn (for SNVs) using the older version of Mutect (data not shown).

In our study we evaluated the impact of several factors on the list of called mutations. The effect of alignment on mutation calling has been recently reported as minor [20]. We report here concordant results in terms of overall performances; however, we clearly show that most of the false positives are a consequence of misalignment. Indeed, selecting the somatic mutations identified by the same caller but after alignment with two different algorithms allowed us to remove around 50% of false positives with a minimal loss (~1%) in sensitivity. A bigger impact was the base quality recalibration step; when not applied, it causes a huge number of false positives. Interestingly, in a recent comparison of whole genome sequencing pipelines, only 5 of the 18 involved groups applied a base quality recalibration step [5].

Overall, Mutect2 outperformed Strelka in terms of sensitivity, particularly at lower VAFs, although showing a tendency for higher FPR/Mb. Both tools benefited from an adjustment of default filtering thresholds, an aspect often overlooked in previous reports. Importantly, we introduced a T/N ratio as an additional filtering criterion that, in combination with a more relaxed threshold for the alternative allele in the matched normal, allowed an increase of up to 9% sensitivity in Mutect2 calls whilst reducing the FPR/Mb. As a cautionary note, some of the threshold applied in the filtering optimisation step might be in some way dependent of coverage, library preparation or sequencing platform and might not be generalised, but we expect the ITC approach here proposed to be generalisable and valid even when picking different tools. We estimated that the use of the ITC approach approximately doubles the required CPU time compared with a single aligner/single caller approach, and extra storage is temporarily required for the additional bam file to be generated. However, we note that the use of high performance computing and parallelisation is common practice and would minimize this drawback. Indeed, the observed increase in performance far outweighs any drawbacks secondary to increased computing resources required.

Our study clearly indicates the importance and advantages of having a benchmark dataset to test somatic mutation calling pipelines and quantitatively measure their performance. Therefore, the dilution series herein generated represent a valuable resource that we are making publicly available through the precisionFDA platform (https://precision.fda.gov/).

The combination of multiple callers has been previously suggested, but it has involved either taking the intersection or the union of them, drastically losing sensitivity in the first case (in particular when one tool performs worse than the other) or hugely increasing the number of false positives in the second. Here we propose a two-step strategy allowing us to merge the calls from two different tools whilst keeping the FPR/Mb low.

## Conclusions

Identification of somatic SNVs and indels in WES data has been suboptimal. Here we propose a computational approach based on the combination of two aligners and two mutation callers to increase the sensitivity whilst controlling for the false positive rate. We also provide a benchmark dataset based on the platinum genome NA12878 to objectively test the performance of any bioinformatic pipeline for the identification of somatic mutations.

## Additional files

Additional file 1: NA12878 dilution series. (XLSX 36 kb)

Additional file 2: Figures S1–S11. All supplementary figures. (PDF 2333 kb)

Additional file 3: List of false positive SNVs and indels using the ITC approach. (XLSX 43 kb)

Additional file 4: VEP annotation for mutations called using the ITC approach and not by the second best (Mutect2/Novocraft) in AB551 breast cancer sample. (XLSX 143 kb)

## Abbreviations

FPR/Mb: False positive rate per megabase
Indels: Small insertions and deletions
ITC: Intersect-then-combine
NGS: Next generation sequencing
QSI_NT: Quality score reflecting the joint probability of a somatic variant (indel) and NT
QSS_NT: Quality score reflecting the joint probability of a somatic variant (SNV) and NT
SNV: Single nucleotide variant
VEP: Variant effect predictor
WES: Whole exome sequencing
