# Supplementary material for: Intersect-then-combine approach: improving the performance of somatic variant calling in whole exome sequencing data using multiple aligners and callers
Source: Genome Med. 2017 Apr 18;9:35. doi: 10.1186/s13073-017-0425-1 (PMC5394620; doi:10.1186/s13073-017-0425-1)

Supplementary figure legends

Figure S1 - Effect of Novoalign alignment score threshold
Effect of Novoalign t parameter on alignment time (A) and alignment efficiency (B).

Figure S2 - NA12878 dilution experiment quality control
(A) Coverage distribution for the 12 samples in the NA12878 dilution series. Each line represent the percentage of target regions having the indicated coverage or higher. (B) VAF distribution of heterozygous and homozygous platinum calls in the NA12878 dilution series. The title of each plot reports the sample ID, the percentage of NA12878 DNA and the percentage of NA11840 DNA respectively. Dashed lines represent the median VAF for heterozygous (pink) and homozygous (cyan) SNVs. Bam files from the Novocraft pipeline were used.

Figure S3 - Observed VAFs for platinum calls in sample 1 (100% NA12878) and sample 12 (100% NA11840)
(A) VAF versus depth of coverage plot for 9968 NA12878 platinum SNVs. (B) Histogram of VAF distribution for 9968 NA12878 platinum SNVs in the NA11840 sample. Bam files from the Novocraft pipeline have been used.

Figure S4 - False positives in the NA12878 dilution series
(A) FPR/Mb in the NA12878 dilution series using Strelka in combination with each alignment pipeline. All calls not in the list of 9968 platinum SNVs were here on considered as false positives. The number of false positive has an unexpected association with the percentage of NA12878, suggesting that most of them are actually real mutations not in the platinum calls, possibly acquired by the cell line during *in vitro* culturing.
(B, C) Intersection between false positives in the 100% and 80% NA12878 samples (top) and 0.5% and 0.2% NA12878 samples (bottom) using Strelka in combination with BWA/GATK pipeline (B) or Novocraft pipeline (C). The fact that most of false positives in the 100% and 80% NA12878 sample overlap and none of them overlap in the diluted samples justified our choice to estimate the FPR/Mb in the 0.2% NA12878 sample.

Figure S5 - Reason of rejection in Mutect2 for false negative calls
For the subset of false negative calls in Mutect2 after BWA/GATK, we summarized how many calls failed each filter in the 100% NA12878 sample (A) and 10% NA12878 sample (B). The same information is reported in C and D for Mutect2 after Novocraft alignment.

Figure S6 - Adjustment of filtering parameters in BWA/GATK/Mutect2 SNV calls
Sensitivity (top) and FPR/Mb (bottom) variations in the 100% NA12878 sample after changing the percentage of tolerated alternative allele in the matched normal (A). In addition to each percentage threshold we applied a threshold to the tumour/normal VAF ratio from 0 to 10 (x-axis), with lower values associated with higher FPR/Mb. (B) As in (A) but in the 10% NA12878 sample. (C) Sensitivity and FPR/Mb variations in the 100% NA12878 sample as a function of the tumour LOD (log odds) score threshold applied. (D) As in C but in the 10% NA12878 sample.

Figure S7 - Adjustment of filtering parameters in Novocraft/Mutect2 SNV calls
(A) Sensitivity (top) and FPR/Mb (bottom) variations in the 100% NA12878 sample after changing the percentage of tolerated alternative allele in the matched normal. For each percentage threshold we also applied a threshold to the tumour/normal VAF ratio from 0 to 10 (x-axis), with lower values associated with higher FPR/Mb. (B) As in A but in the 10% NA12878 sample. (C) Sensitivity and FPR/Mb variations in the 100% NA12878 sample as a function of the tumour LOD threshold applied. (D) As in C but in the 10% NA12878 sample.

Figure S8 - Adjustment of filtering parameters in Strelka SNV calls
(A) Sensitivity and FPR/Mb variations in the 100% NA12878 sample after changing the QSS_NT threshold. Values obtained using Tier 1 or both Tier 1 and Tier 2 calls are reported. Raw data have been aligned using the BWA/GATK pipeline. (B) As in A but in the 10% NA12878 sample. (C-D) Same analysis in A-B but on data aligned using Novocraft.

Figure S9 - Adjustment of filtering parameters in Strelka Indels calls
(A) Sensitivity and FPR/Mb variations in the 100% NA12878 sample after changing the QSI_NT threshold. Values obtained using Tier 1 or both Tier 1 and Tier 2 calls are reported. Raw data have been aligned using the BWA/GATK pipeline. (B) As in A but in the 10% NA12878 sample. (C-D) Same analysis in A-B but on data aligned using Novocraft.

Figure S10 - Performance of the intersect-then-combine approach, heterozygous mutations only
(A) Sensitivity in identifying SNVs after applying the ITC strategy compared with performance using each single alignment pipeline in combination with each mutation caller. Only SNVs heterozygous in NA12878 sample are included. (B) Sensitivity in identifying 'somatic' Indels after applying the ITC strategy compared with performance using each single alignment pipeline in combination with each mutation caller. Only Indels heterozygous in NA12878 sample are included.

Figure S11 - IGV view of representative false positive SNVs and Indels
Three bam files have been loaded: 0.2% NA12878 (where FPR/Mb was estimated), 100% NA11840 (used as normal sample) and 100% NA12878 to verify that the mutation was not present in the undiluted sample. (A) chr3:151055698 C>A false positive SNV. (B) chr22:21983617 T>TCCCA false positive insertion.

Figure S1 - Effect of novoalign alignment score threshold t

A

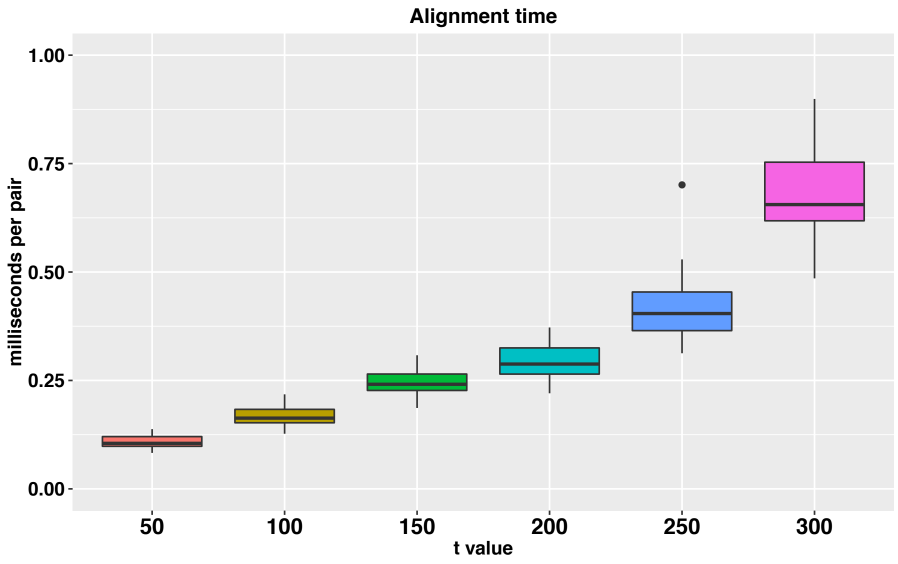

B

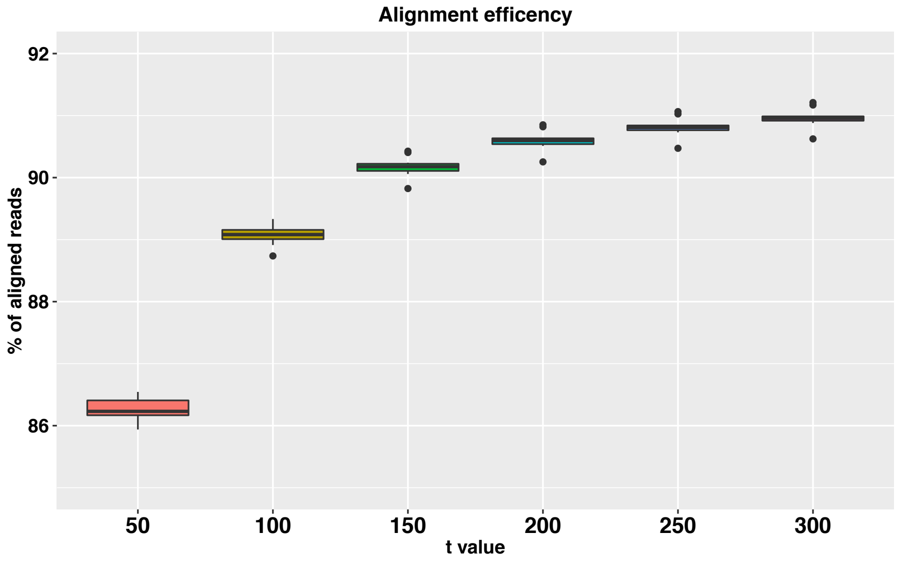

Figure S2 – NA12878 dilution experiment quality control

A

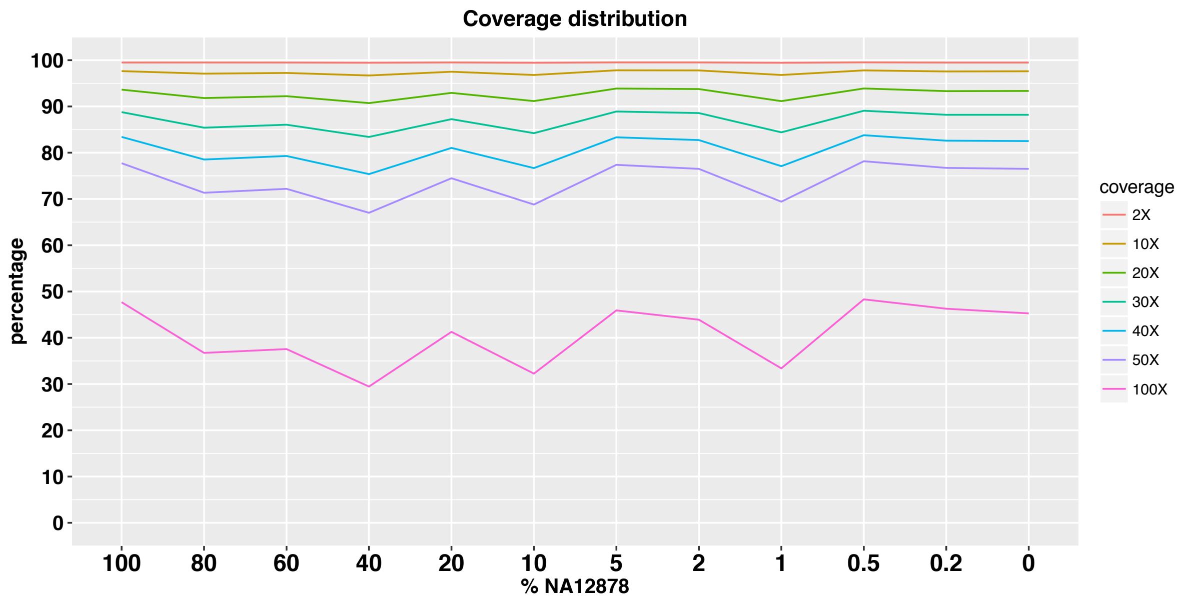

B

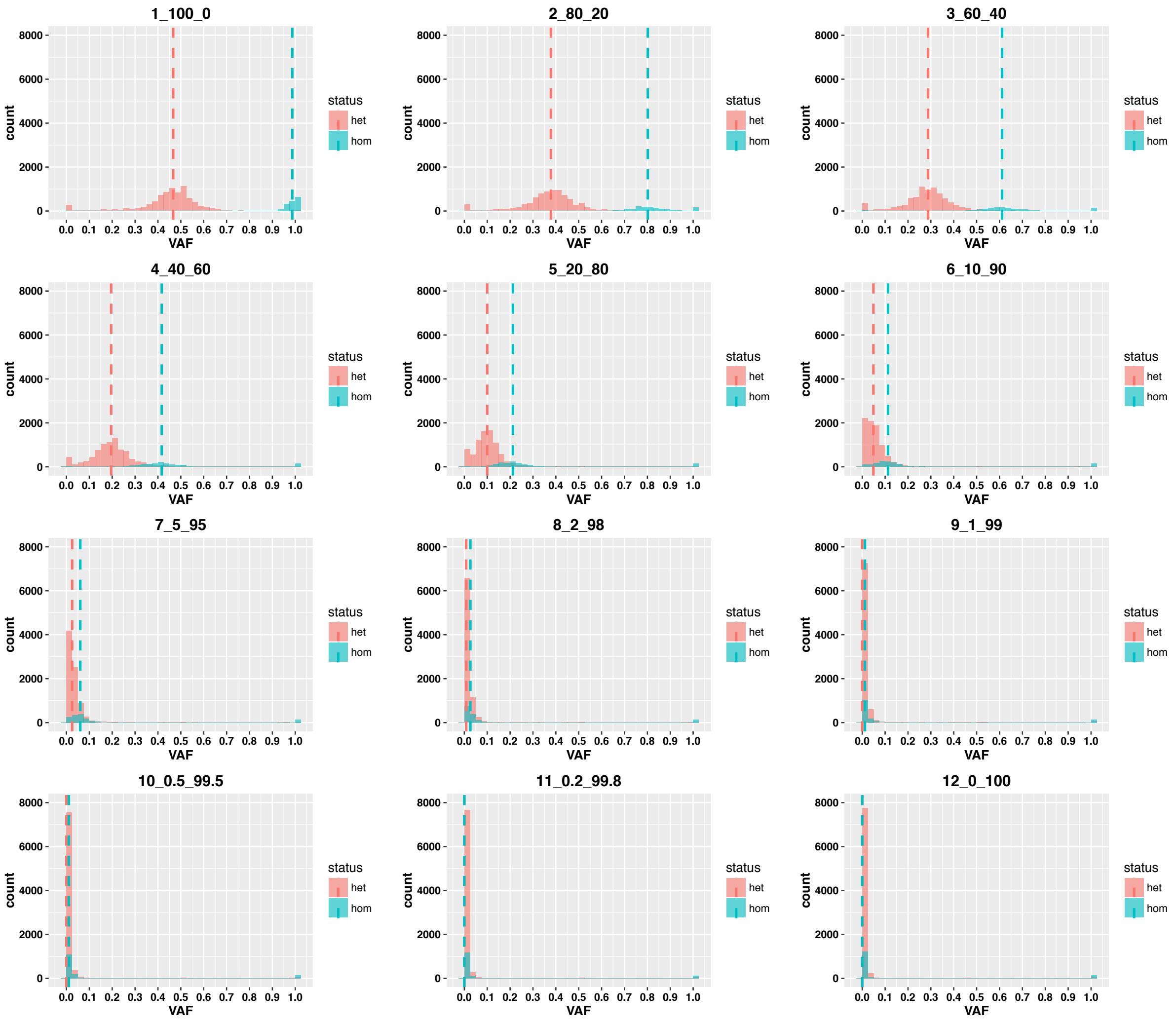

Figure S3 – Observed VAFs for platinum calls in sample 1 (100% NA12878) and sample 12 (100% NA11840)

A

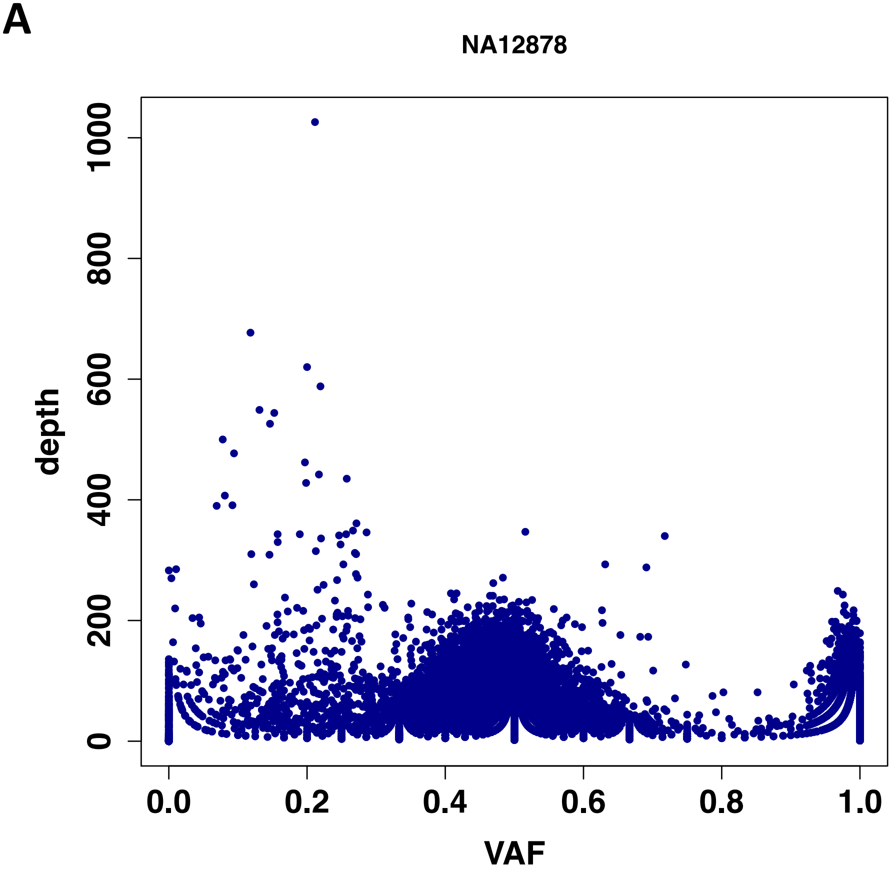

B

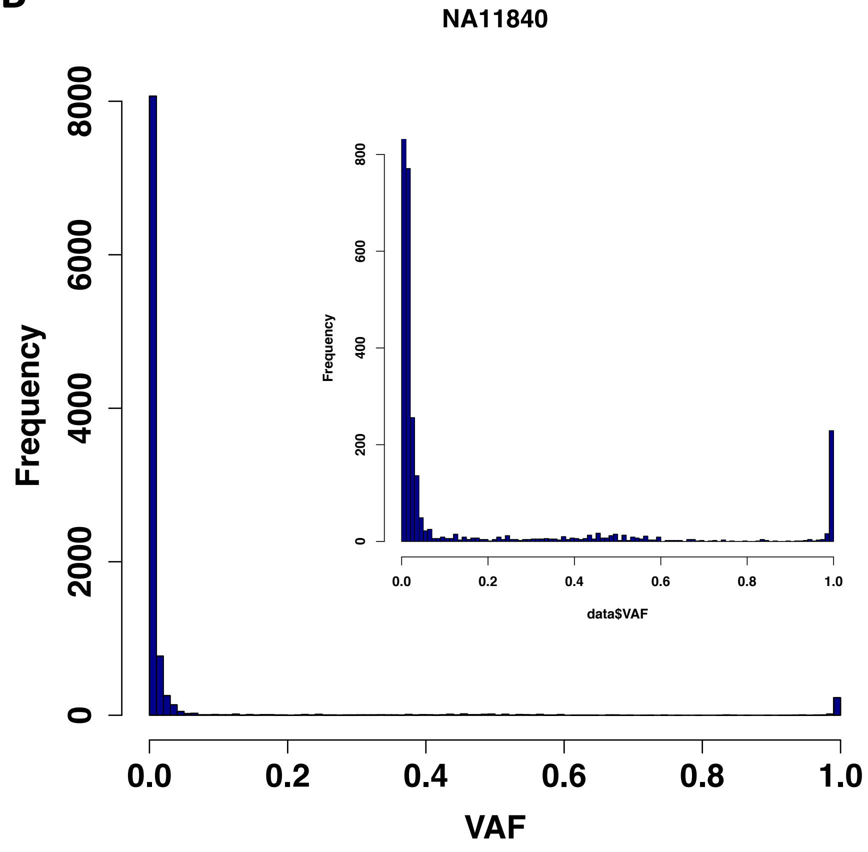

Figure S4 – False positives in NA12878 dilution series

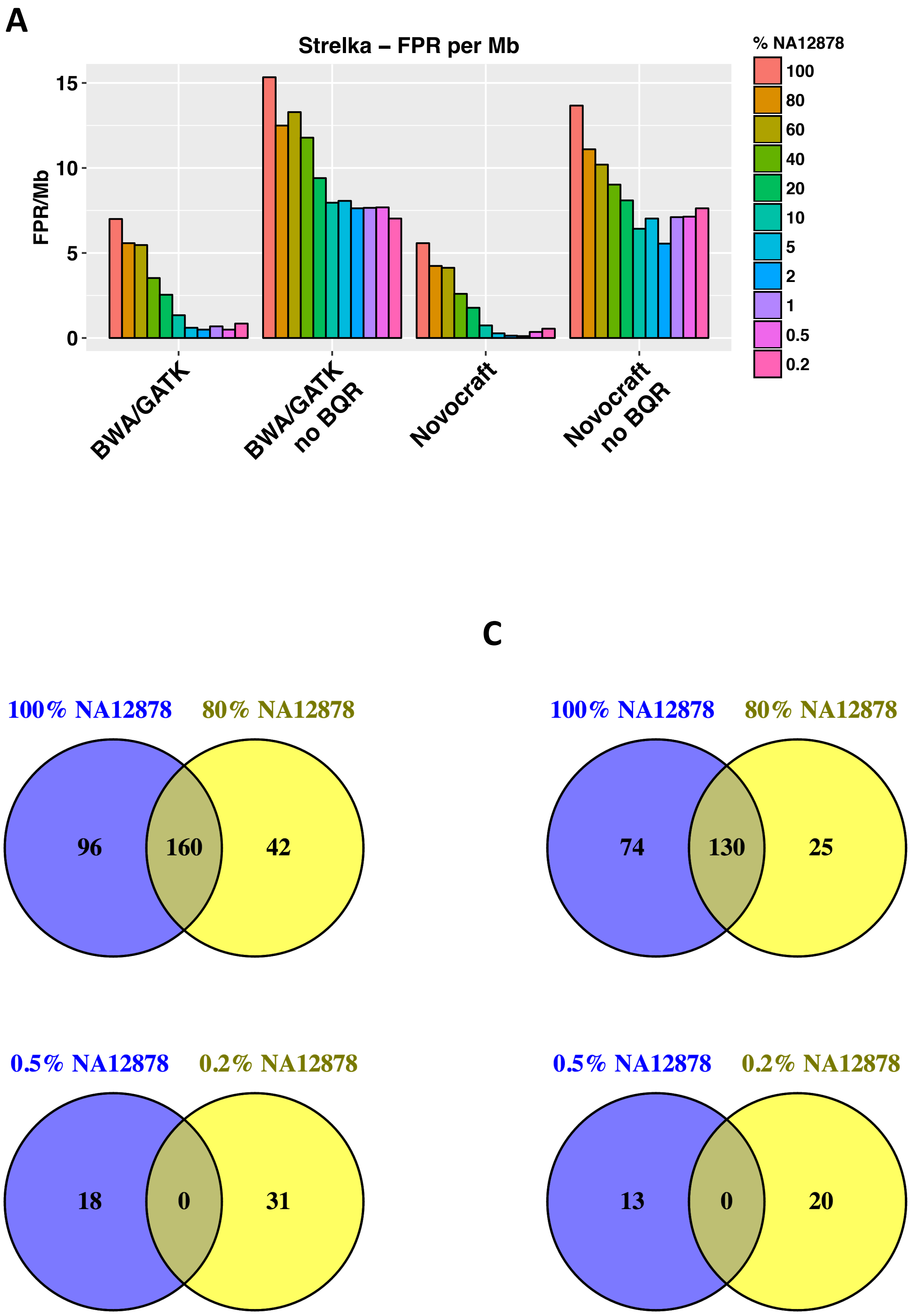

Figure S5 - Reason of rejection in Mutect2 for false negative calls

A

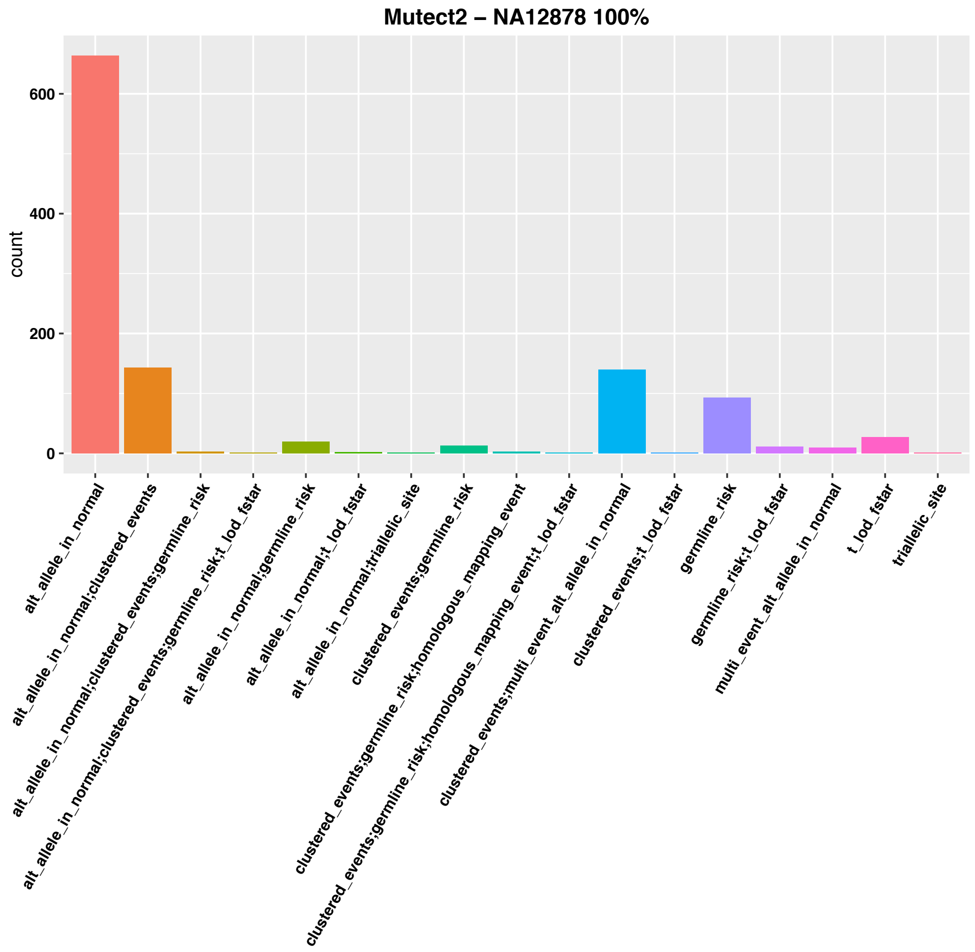

B

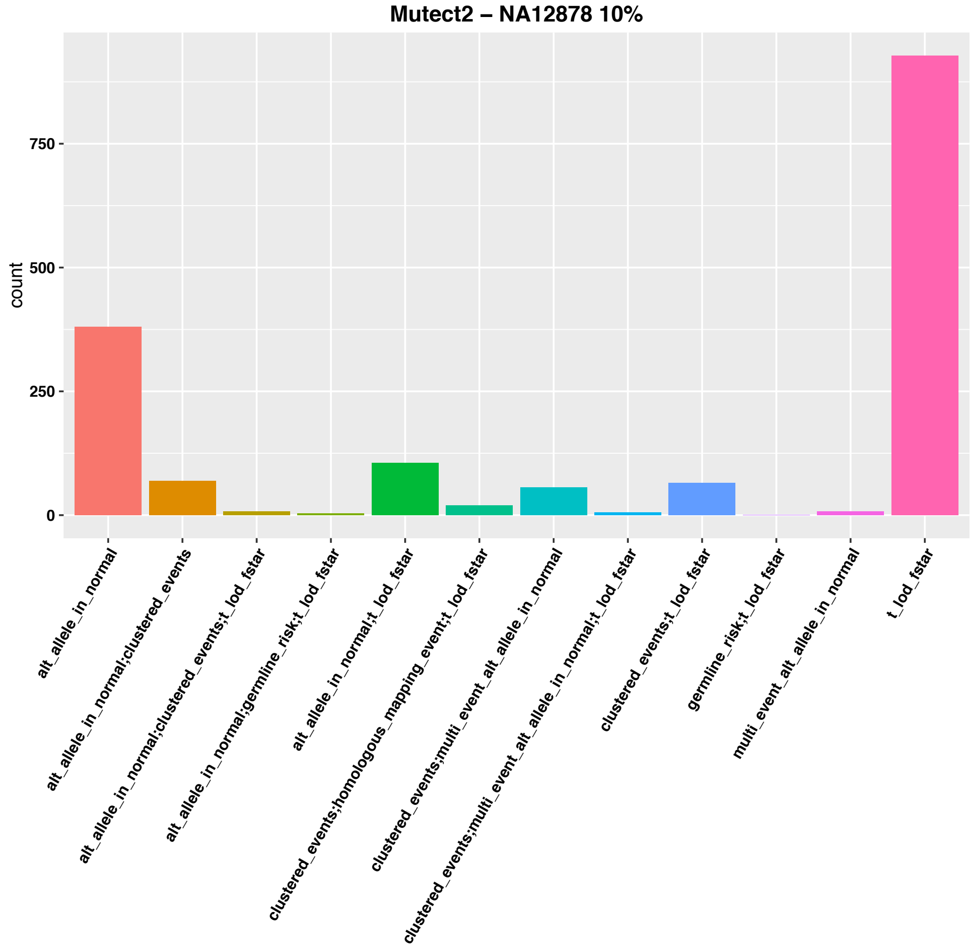

C

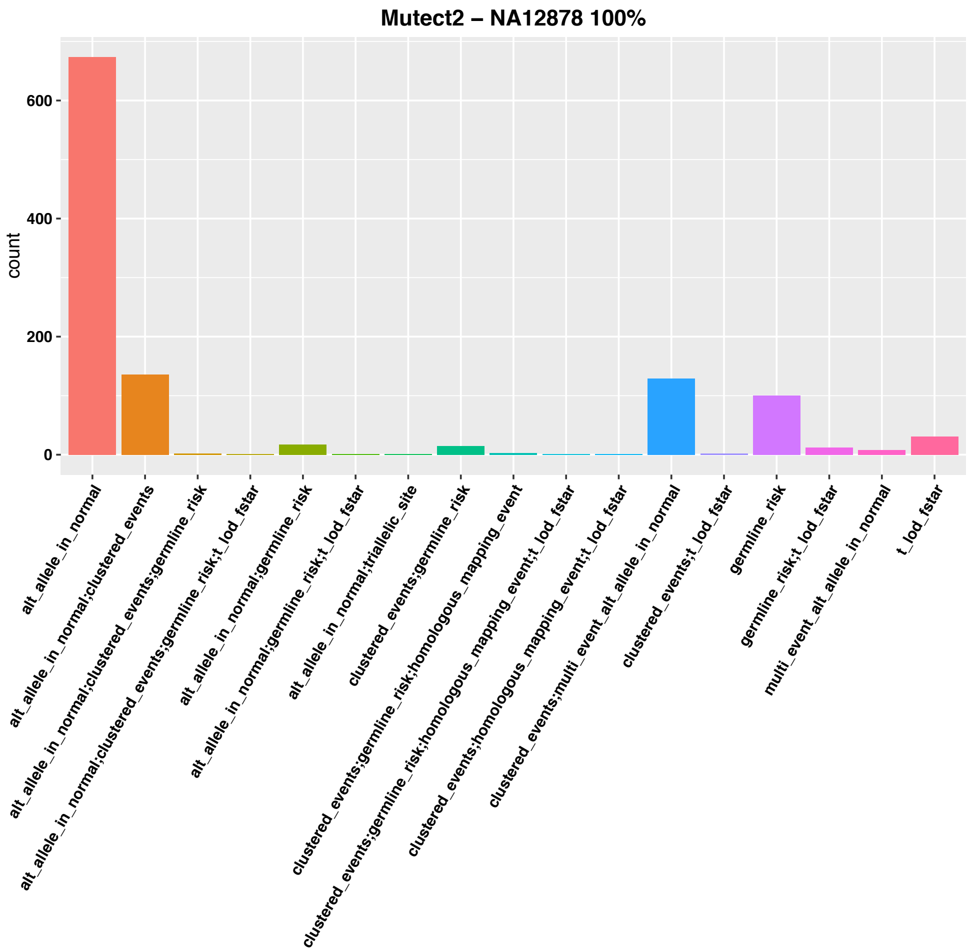
Mutect2 – NA12878 100%
count
600
400
200
0
alt_allele_in_normal
alt_allele_in_normal;clustered_events
alt_allele_in_normal;clustered_events;germline_risk
alt_allele_in_normal;clustered_events;germline_risk;t_lod_fstar
alt_allele_in_normal;germline_risk
alt_allele_in_normal;germline_risk;t_lod_fstar
alt_allele_in_normal;triallelic_site
clustered_events;germline_risk
clustered_events;germline_risk;homologous_mapping_event
clustered_events;germline_risk;homologous_mapping_event;t_lod_fstar
clustered_events;homologous_mapping_event;t_lod_fstar
clustered_events;multi_event_alt_allele_in_normal
clustered_events;t_lod_fstar
germline_risk
germline_risk;t_lod_fstar
multi_event_alt_allele_in_normal
t_lod_fstar

D

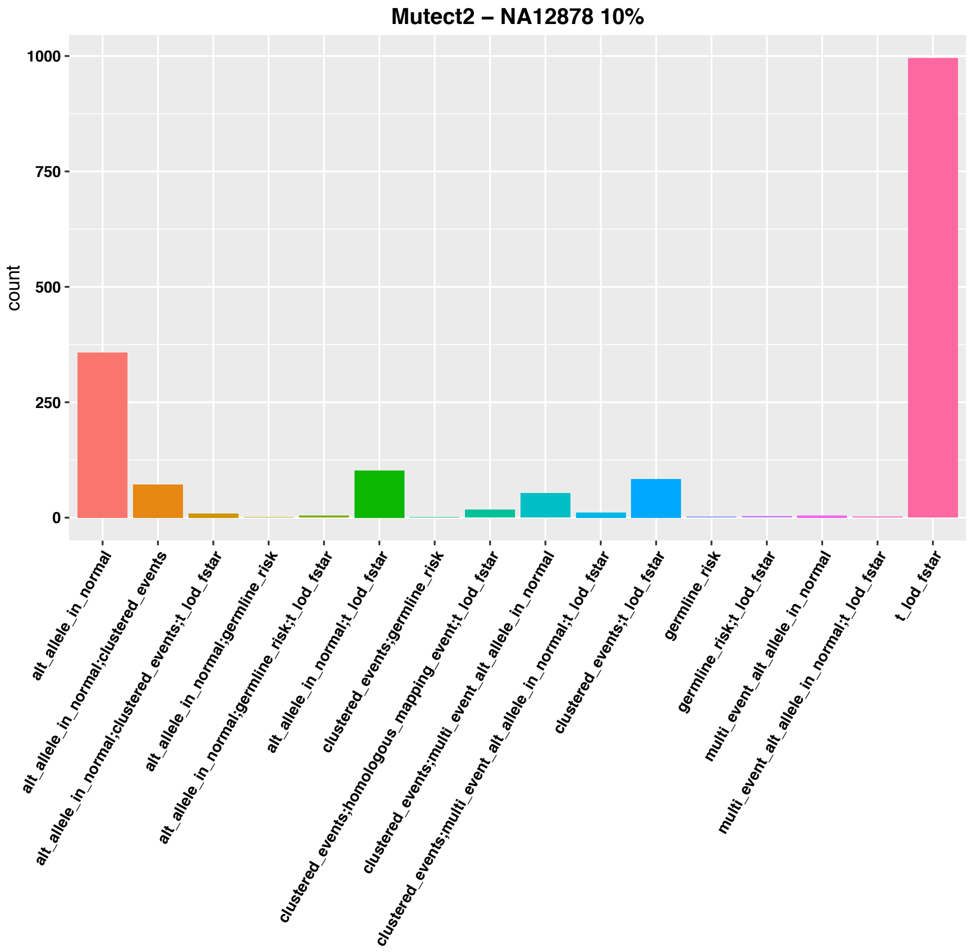
Mutect2 – NA12878 10%
count
1000
750
500
250
0
alt_allele_in_normal
alt_allele_in_normal;clustered_events
alt_allele_in_normal;clustered_events;t_lod_fstar
alt_allele_in_normal;germline_risk
alt_allele_in_normal;germline_risk;t_lod_fstar
alt_allele_in_normal;t_lod_fstar
clustered_events;germline_risk
clustered_events;homologous_mapping_event;t_lod_fstar
clustered_events;multi_event_alt_allele_in_normal
clustered_events;multi_event_alt_allele_in_normal;t_lod_fstar
clustered_events;t_lod_fstar
germline_risk
germline_risk;t_lod_fstar
multi_event_alt_allele_in_normal
multi_event_alt_allele_in_normal;t_lod_fstar
t_lod_fstar

Figure S6 - Adjustment of filtering parameters in BWA/GATK/Mutect2 SNV calls

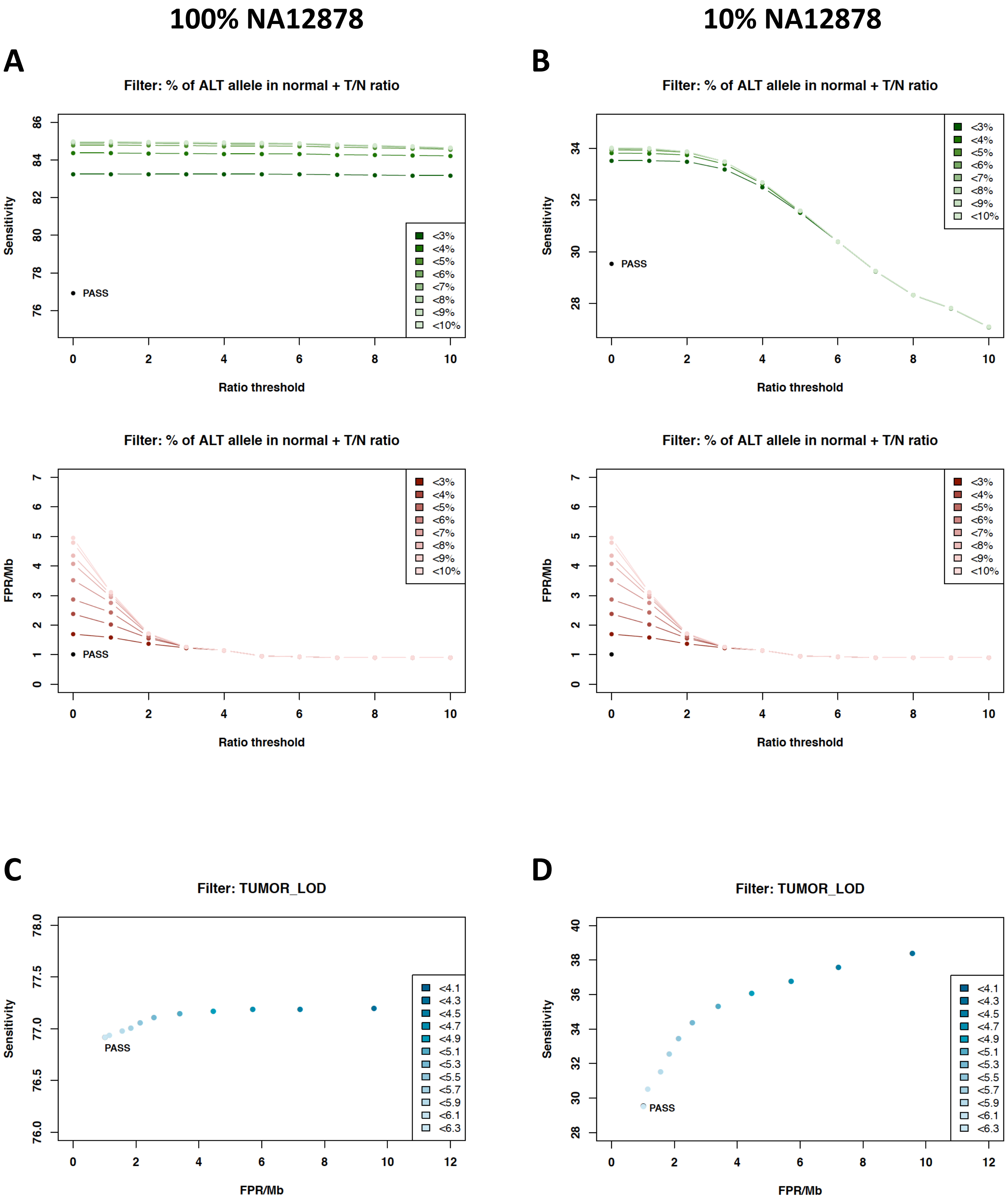

Figure S7 - Adjustment of filtering parameters in Novocraft/Mutect2 SNV calls

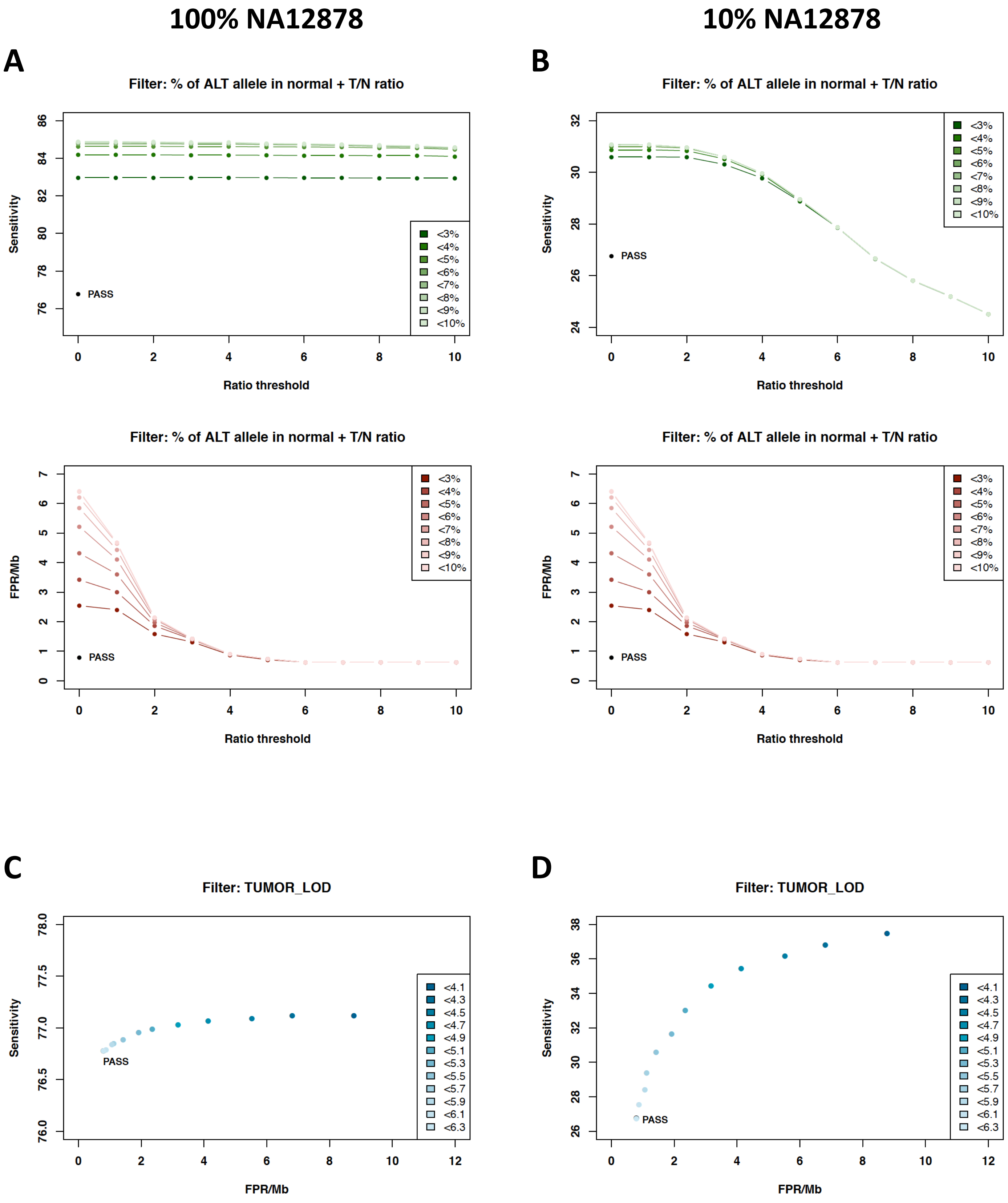

Figure S8 - Adjustment of filtering parameters in Strelka SNV calls

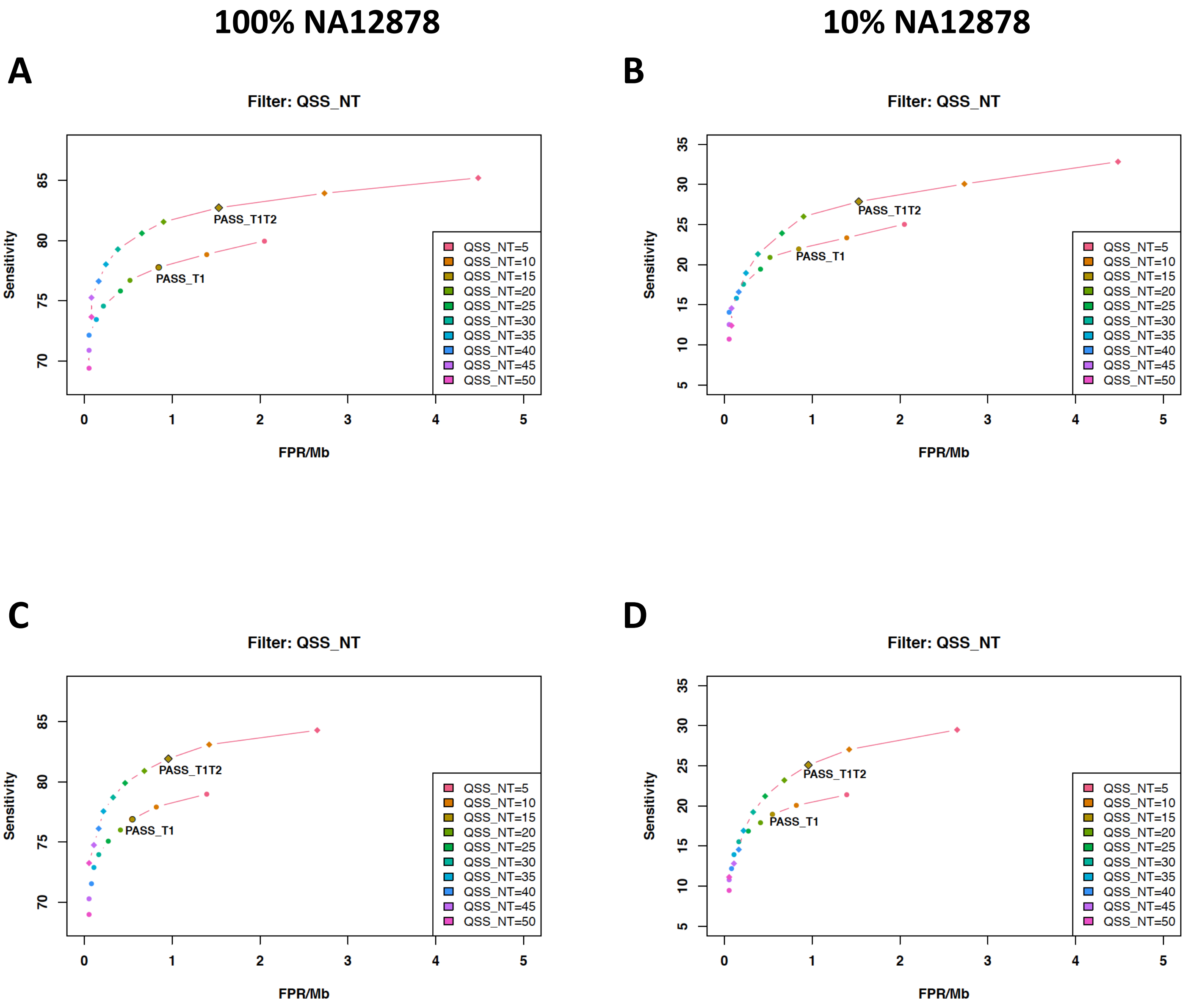

Figure S9 - Adjustment of filtering parameters in Strelka Indels calls

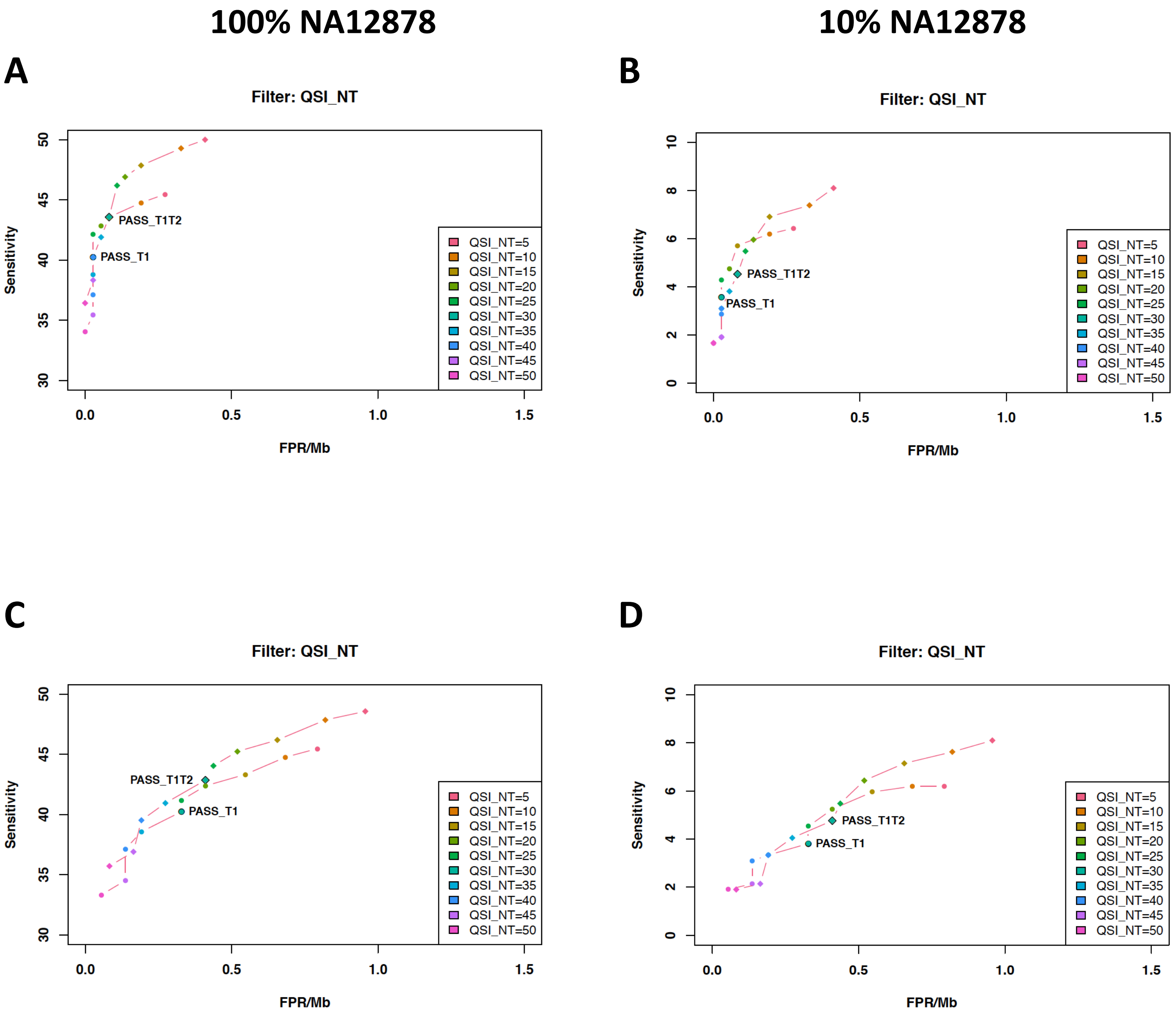

Figure S10 - Performance of the intersect-then-combine approach, heterozygous mutations only

A

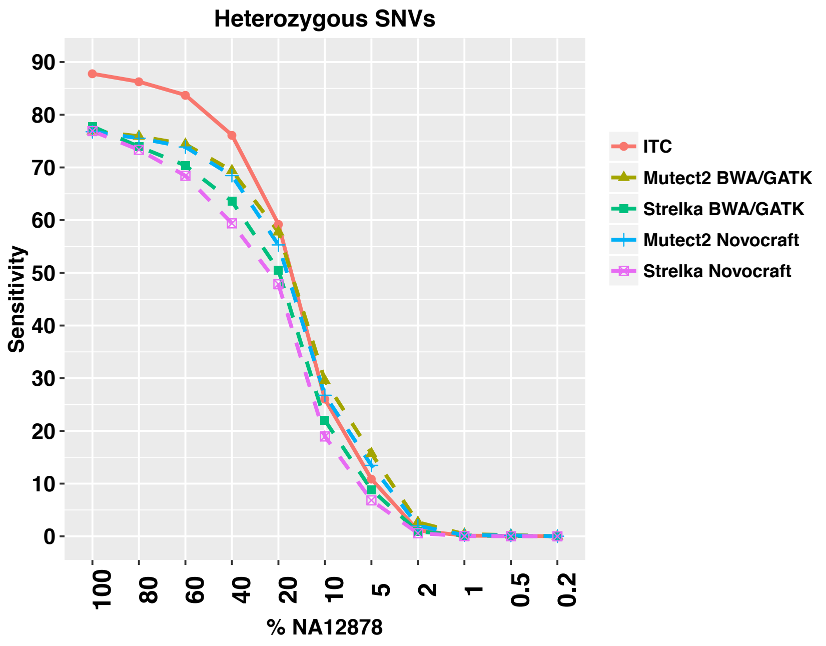

B

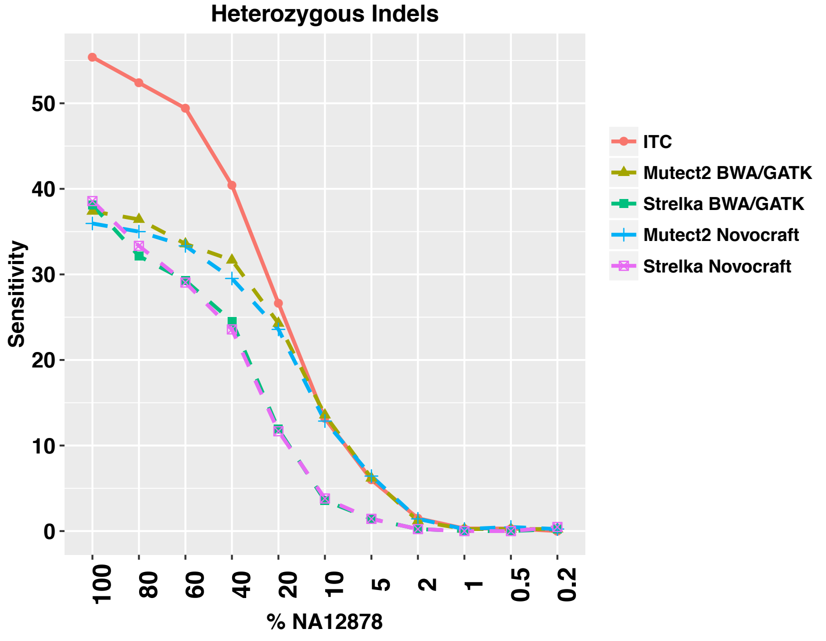

Figure S11 – IGV view of representative false positive SNVs and Indels

A

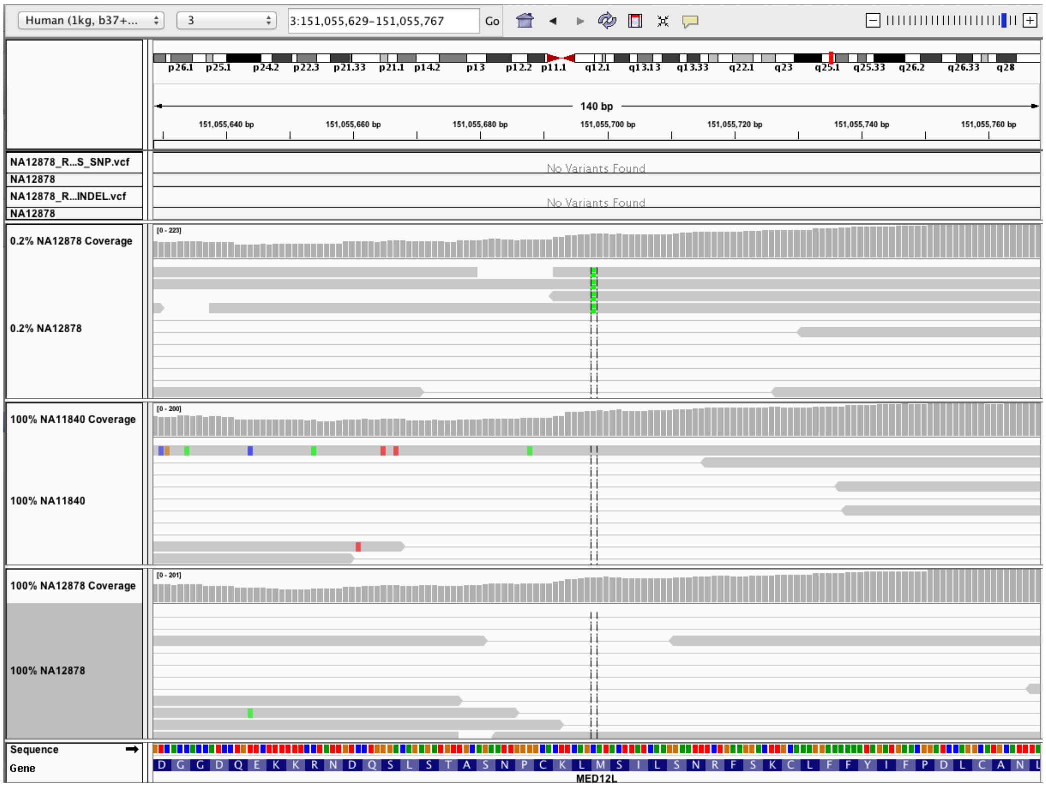

B

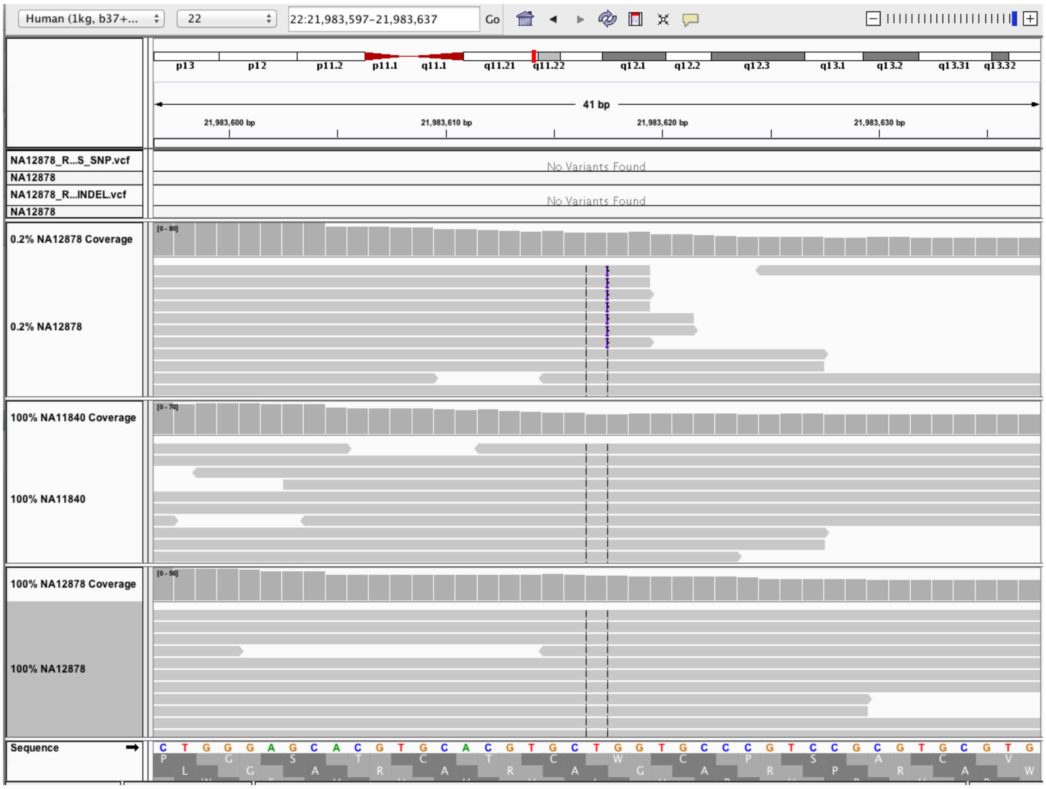

Supplement: Supplementary file 2 — All supplementary figures. (PDF 2333 kb) [file 13073_2017_425_MOESM2_ESM.pdf]
